# DECAL: A Reconfigurable Monolithic Active Pixel Sensor for Tracking and Calorimetry in a 180 nm Image Sensor Process

**DOI:** 10.3390/s22186848

**Published:** 2022-09-10

**Authors:** Philip Patrick Allport, Seddik Benhammadi, Robert Ross Bosley, Jens Dopke, Lucian Fasselt, Samuel Flynn, Laura Gonella, Nicola Guerrini, Cigdem Issever, Kostas Nikolopoulos, Ioannis Kopsalis, Peter Philips, Tony Price, Iain Sedgwick, Giulio Villani, Matt Warren, Nigel Watson, Hannsjorg Weber, Alasdair Winter, Fergus Wilson, Steven Worm, Zhige Zhang

**Affiliations:** 1 School of Physics and Astronomy, University of Birmingham, Edgbaston, Birmingham B15 2TT, UK; 2 STFC Rutherford Appleton Laboratory, Harwell Campus, Didcot OX11 0QX, UK; 3 Institute for Physics, Humboldt University of Berlin, Newtonstrasse 15, D-12489 Berlin, Germany; 4 Deutsches Elektronen-Synchrotron DESY, Platanenallee 6, D-15738 Zeuthen, Germany; 5 Department of Physics and Astronomy, University College London, Gower Street, London WC1E 6BT, UK

**Keywords:** MAPS, CMOS, tracking, calorimetry, particle physics, ECAL

## Abstract

In this paper, we describe DECAL, a prototype Monolithic Active Pixel Sensor (MAPS) device designed to demonstrate the feasibility of both digital calorimetry and reconfigurability in ASICs for particle physics. The goal of this architecture is to help reduce the development and manufacturing costs of detectors for future colliders by developing a chip that can operate both as a digital silicon calorimeter and a tracking chip. The prototype sensor consists of a matrix of 64 × 64 55 μm pixels, and provides a readout at 40 MHz of the number of particles which have struck the matrix in the preceding 25 ns. It can be configured to report this as a total sum across the sensor (equivalent to the pad of an analogue calorimeter) or the sum per column (equivalent to a traditional strip detector). The design and operation of the sensor are described, and the results of chip characterisation are reported and compared to simulations.

## 1. Introduction

Calorimetry is an important technique for the determination of the energy of incident particles in a sub-detector [1,2,3,4], and it can be broadly divided into Electro-Magnetic Calorimetry (EMCAL or ECAL) [5] and Hadronic Calorimetry (HCAL) [6]. EMCAL devices detect charged particles, and neutral particles are detected in HCAL detectors. Both operate on the principle of stopping incident particles in some form of absorber material. This generates a signal which can be measured, and also a shower of secondary particles, which stop in later layers of the calorimeter, and can then be measured at this point. Combining these measurements allows the energy of the initial particle to be determined. Since silicon is a suitable detector material for ionising particles, it can be used as a detector layer in an ECAL device. A small fraction of electromagnetic calorimeters are silicon-based devices, with the largest example under construction being a detector for CMS [7]. They are typically constructed from layers of a dense, high atomic number, absorber material (such as lead or tungsten) interleaved with silicon as the detecting material. Particles incident on the absorber initiate showers of lower energy particles, which deposit energy in the detecting layers. The total charge deposited in each detector layer is then summed and read out. By performing this operation for each detector layer, the energy of the initial particle can be determined.

However, it has been suggested that a digital approach can lead to higher energy resolution [8,9] and potentially take better advantage of low-cost commercial imaging sensor technologies. In contrast to an analogue calorimeter, which counts the total deposited energy in a given detector volume, a digital electromagnetic calorimeter is segmented and counts the total number of particles passing through the volume. This can potentially provide better energy resolution at a lower cost. Digital calorimetry has been studied for several possible future applications [10,11], a demonstration calorimeter using an existing, non-optimised chip has been constructed [12,13] and some research has been undertaken into chips designed specifically for this application [14,15,16].

Particle detectors also make considerable use of strip sensors [17,18,19,20,21]. These are detectors in which a strip of silicon detector material is bonded at one end to an Application Specific Integrated Circuit (ASIC) for readout, resulting in a long thin detector which can cover large areas. Since the proposed digital calorimeter is also made of silicon, its cost effectiveness can be further improved if it can also be configured to operate as a strip sensor, which could also serve in outer tracking and pre-shower layers. This is because the cost of producing ASICs, especially for low-volume applications such as particle physics detectors, has for some time been dominated by the development costs, and this is a trend that is only becoming more pronounced as transistor technology improves and the devices are manufactured in smaller but more complex technologies [22]. In this paper, therefore, we describe DECAL, a Monolithic Active Pixel Sensor (MAPS) designed for use as both a digital electromagnetic calorimeter and a tracker. This development has the overall goal of accessing the good performance of a digital calorimeter, whilst achieving high cost effectiveness through re-use of the chip in different applications.

## 2. Physics Background and Specifications

The major advantage of a digital calorimeter over an analogue approach is that it counts the number of incident particles rather than summing the total deposited charge. This removes the impact of fluctuations in the deposited charge on the energy resolution of the calorimeter. Studies have already been published evaluating this performance [23,24,25], and in [26] an energy resolution of $\frac{16.8\%}{\sqrt[{}]{E}}\;⊕$ 0.6 % was demonstrated for a sensor geometry very similar to that considered here.

**Figure 1:**
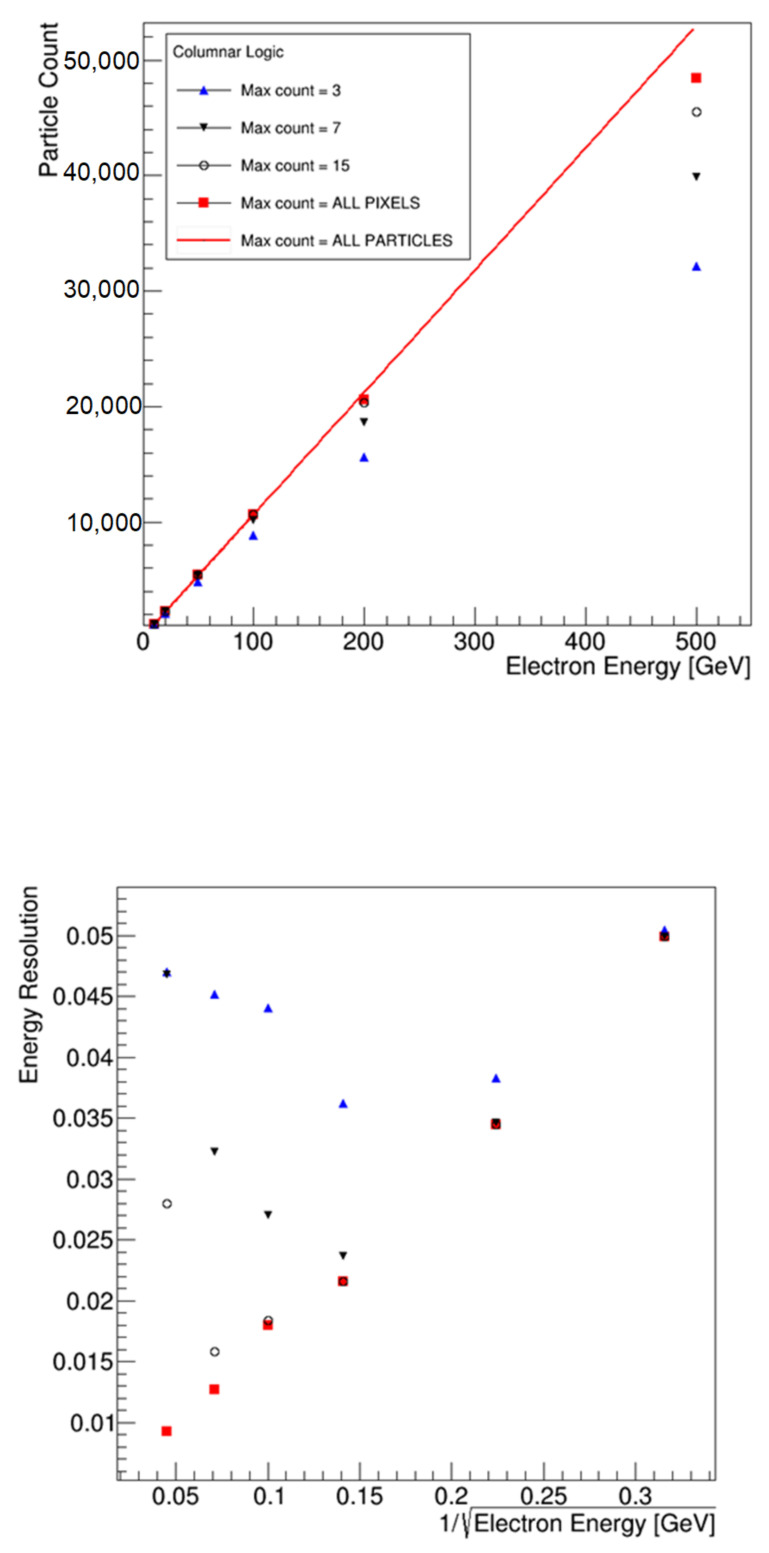
Study of achievable energy resolution for varying limits imposed on the maximum particle count per column. The upper plot shows the total number of particles counted for a range of detector configurations for the same incident particle energy. The lower plot shows the resulting energy resolution.

However, these studies assume a reasonably ideal detector, which can count all incident hits up until the point where an individual pixel experiences multiple hits (pile-up) and cannot separate them. Due to the practical requirements of implementing the counting mechanism on-chip however, it is likely that there will be some limit on the number of hits that can be counted before the count circuitry is saturated. This is likely to be much lower than the point at which pixels begin to experience pile-up. To inform the design and establish the maximum count rate needed to avoid limiting performance, studies of the effect of this limit were undertaken. To achieve this, a Geant4 simulation model of a calorimeter was developed which consisted of concentric layers of tungsten and silicon. The silicon layers were those used for detection. The simulation was run with incident particles of varying energies up to 500 GeV. The goal of the simulations was to establish what the lowest maximum count level per column could be in order to avoid a significant loss of energy resolution. Figure 1 shows the output of these simulations.

As a baseline, the total number of particles incident on the silicon was counted. This is indicated by the red line marked “ALL PARTICLES”. Then, the number of pixels generating a signal in silicon over a given detection threshold was calculated (“ALL PIXELS”). At higher energies, this deviates from the absolute particle count due to multiple particles being incident upon a single pixel in the dense cores of the particle shower. The impact of imposing a limit on the maximum sum value in a column was investigated by limiting the number of pixels in a readout cycle per column (the traces labelled “Max Count”). It can be seen that for a reduced number of hits allowed per column, the count becomes increasingly non-linear and deviates further from the pixel count at higher energies. This is due to the under counting of the number of particles in the shower. It is, therefore, essential to have the largest number of hits allowed per column. The consequences of the undercounting are observed in the energy resolution plot for the same conditions, where it can be seen that a minimum of 15 pixels per column are required to obtain the same results as a conventional calorimeter up to 200 GeV. Therefore, a maximum count of 15 hits per column was selected. This implies a 4-bit counting bus per column.

As well as providing a complete sum of hits over the sensor for calorimetry purposes (referred to as “Pad Mode”), the sensor must also be capable of operating as the equivalent of a strip sensor for tracking, by instead providing the total number of hits in a column for every column (“Strip Mode”). It is not necessary to provide the full 4 bit count per column in this mode, and in some applications a simple 1-bit hit/no hit output per column would be sufficient. However, it was decided to transmit 2 bits per column in this mode. This is a compromise between providing the maximum amount of useful information, and keeping the data output within manageable limits (since this prototype is not intended to have any zero suppression). To ensure that the user is warned if the chip is saturated, overflow flags should be provided if the maximum count values are exceeded.

Another important specification is the speed of the chip. In this prototype, we target a speed suitable for the existing Large Hadron Collider (LHC). Therefore, the count value must be delivered for every bunch crossing, so the sensor must be capable of calculating and outputting this value every 25 ns.

For both modes, the input referred noise should be <80 $e^{-}$ to allow detection of a 500 $e^{-}$ signal at 6σ. Such a noise level would also ensure that a Minimum Ionising Particle (MIP) signal was well above the noise, and could be detected even as a split event (an event in which an ionising particle impacts the chip on the boundary between several pixels, leading to the resulting charge being spread across several pixels, reducing the signal available for detection by one pixel). Specifications for the chip are summarized in Table 1.

## 3. Sensor Overview and Timing

**Figure 2:**
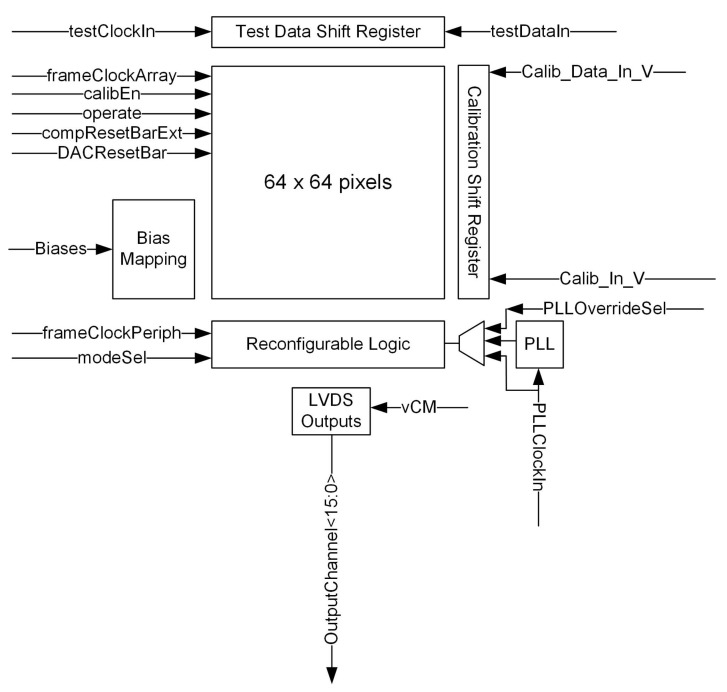
Top-Level plan of the chip.

In this section, we provide a description of the sensor design and circuitry created to meet the requirements of the previous section. Figure 2 shows an outline of the sensor design.

The core of the sensor is the pixel array. Pixel design is described in a later section. Within a column, hits from the pixel are summed to provide a per column total to the readout logic at the bottom of the column. This either sums the number of hits across all columns (pad mode) or outputs the number of hits per column (strip mode). The chip also includes a test register. This injects 5 bits of data into the summation scheme (see Section 3.2), which permits the column and periphery circuitry to be tested independently of the pixels.

**Figure 3:**
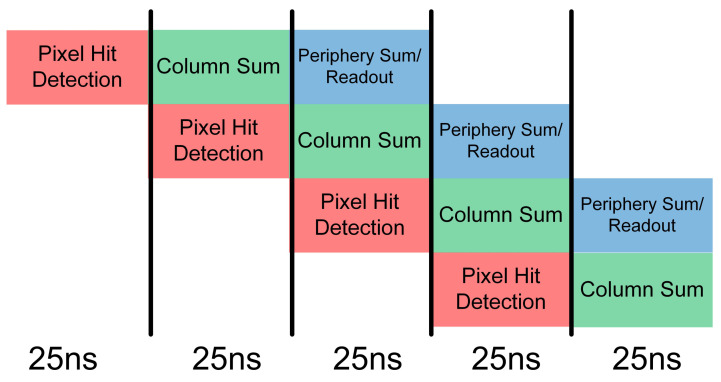
Illustration of the 25 ns chip readout pipeline.

Each of these steps (pixel hit detection, column summation and peripheral readout) requires 25 ns to complete, so the readout is pipelined as shown in Figure 3.

### 3.1. Pixel

**Figure 4:**
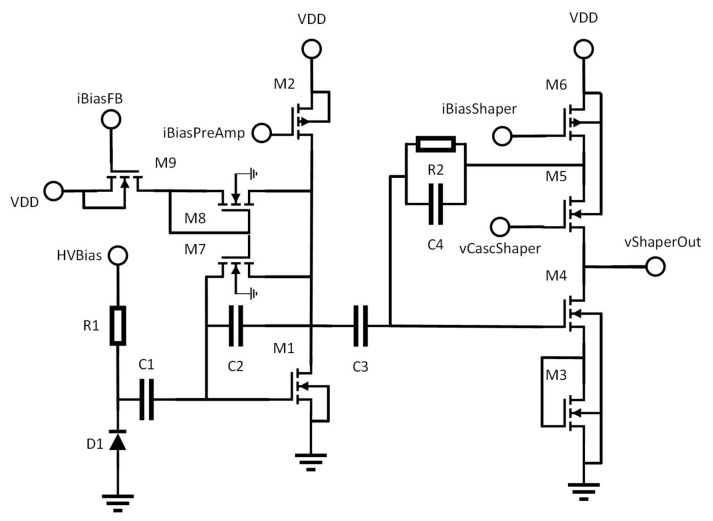
Schematic of the pixel front end.

**Figure 5:**
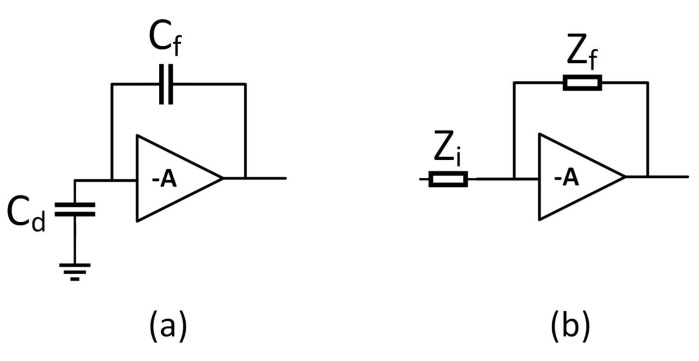
The pixel front end separated into simplified models of the pre-amplfier (**a**) and the shaper (**b**).

We start our description of the sensor by looking at the pixel. Hits are first detected by the analogue front-end, which is a pre-amplifier, shaper, comparator type as shown in Figure 4. This is a standard architecture which is commonly used for pixels of this type [9,15,27,28,29,30,31]. Charge generated in the silicon is collected by the diode D1. To allow this to be biased to the highest possible voltage in order to generate a large depletion region, it is AC-coupled to the remainder of the front-end circuitry through the capacitor C1, and biased through the resistor R1. Transistor M1, in combination with the capacitor C2, forms a charge pre-amplifier, which is biased by the transistor M2. Transistors M7–M9 provide a constant current feedback circuit around C1 to sustain the DC bias of the amplifier. Assuming that C1 is large enough to provide a very low impedance, the M1, C1 section can be treated as a simple charge sensitive amplifier as shown in Figure 5a, where $C_{d}$ is the detector capacitance and $C_{f}$ is the value of the feedback capacitor (C2 in Figure 4). The gain of this section can be shown to be $\frac{1}{C_{f}}$ [32] provided that the voltage gain of the amplifier itself is large. Therefore, $C_{f}$ is kept small relative to the detector capacitance to maximise the gain of the first stage, the overall goal being to boost the signal as much as possible.

The signal from the pre-amplifier is then passed to a shaping amplifier formed by transistors M3–M6. This amplifier shapes the pulse coming from the pre-amplifer and helps to filter out noise, improving the SNR. M6 provides the bias current for the amplifier, M5 is a cascode device which helps to provide some rejection of power supply noise, M4 is the amplifying device, and M3 sets the DC level of the output. A simplified diagram of this is shown in Figure 5b. In this simplified diagram, the impedances $Z_{i}$ and $Z_{f}$ are given by

(1) $$Z_{i}=R_{o}+Z_{3}\;\;\;\;\;Z_{f}=\frac{R_{2}Z_{4}}{R_{2}+Z_{4}}$$

where $R_{o}$ is the output resistance of the pre-amplifier, $Z_{3}$ and $Z_{4}$ are the impedances of $C_{3}$ and $C_{4}$ respectively and $R_{2}$ is as shown in Figure 4. The resulting transfer function of the shaper is

(2) $$\frac{V_{o}}{V_{i}}=\frac{-AZ_{f}}{Z_{f}+Z_{i}+AZ_{i}}$$

which, if *A* is very large, simplifies to

(3) $$\frac{V_{o}}{V_{i}}=-\frac{Z_{f}}{Z_{i}}$$

as might be expected. Replacing the impedances with their full complex expressions gives

(4) $$\frac{V_{o}}{V_{i}}=\frac{sC_{3}}{\left(1+sR_{2}C_{4}\right)\left(1+sR_{o}C3\right)}$$

This is the transfer function of a bandpass filter, and illustrates how the shaper is able to provide a noise filtering function. The values of $R_{2}$, $C_{3}$, $C_{4}$, and to a certain extent $R_{o}$ are chosen to maximise SNR by filtering as much noise as possible whilst maximising pulse height.

**Figure 6:**
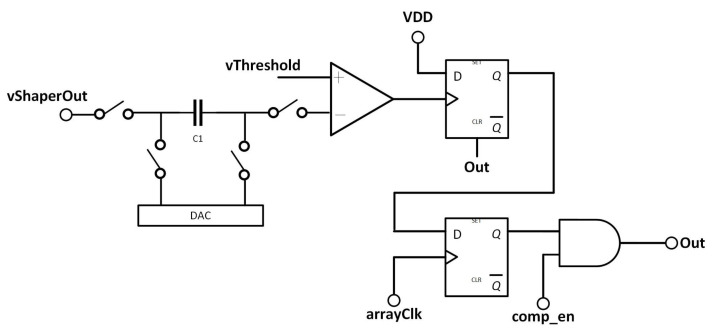
Diagram of the post-front end section of the pixel, showing the placement of the bias DAC, hit register and mask register.

To determine whether or not a hit has occurred, the output of the shaper is then passed to a comparator as shown in Figure 6. This detects a hit if the incoming signal value has passed an externally set detection threshold. Since manufacturing tolerances mean that the shaper output level and the comparator’s offset will vary slightly from pixel to pixel, each pixel effectively experiences a slightly different threshold. To account for this, a capacitor is placed between the shaper output and the comparator input. The voltage on this capacitor can be set using an in-pixel Digital-to-Analogue Converter (DAC). The value applied to the capacitor allows pixel-to-pixel variations to be tuned out, ensuring that all pixels have the same threshold.

**Figure 7:**
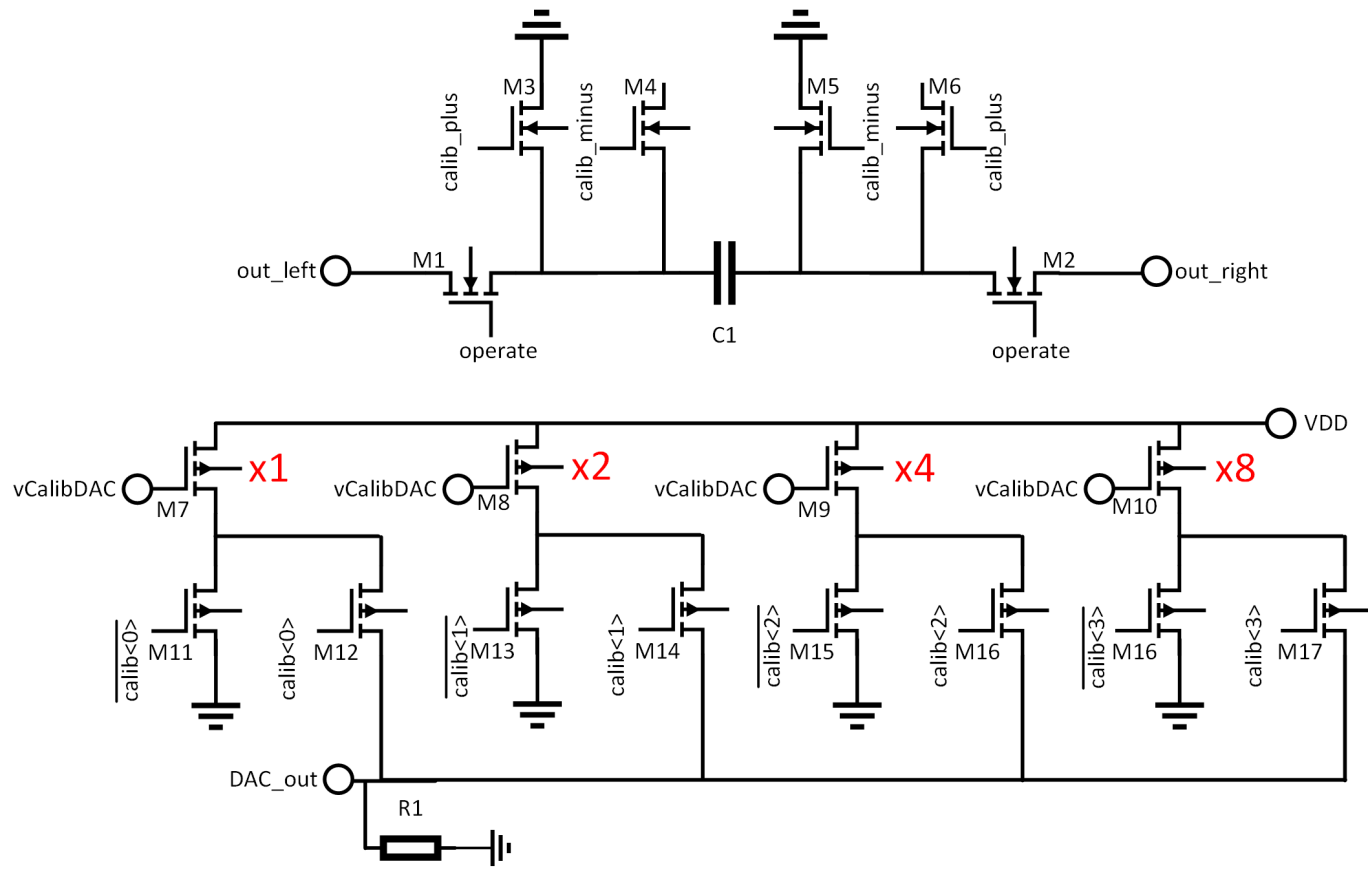
Detailed schematic of the trim DAC and capacitor. For clarity, substrate connections are not shown. All NMOS have their substrates connected to ground, all PMOS to VDD. Relative sizes of the DAC branches are illustrated in red.

Figure 7 shows how the offset is trimmed in more detail, illustrating the internal circuitry of the DAC together with the capacitor C1 on which the offset trim voltage is set. To generate the offset trimming voltage, a binary weighted current DAC (M7–M10) is used to generate a current which is passed through the resistor R1, generating a voltage. Transistors M7–M10 double in size for each bit of the DAC. Since they all receive the same bias voltage, this leads to each transistor generating a current twice the size of the previous branch. The switches M11–M17 are used as the switches for this DAC, switching the current either into R1 or into ground. This is performed in order to ensure that the supply current drawn by the DACs in the many pixels of the array is constant regardless of the trim values that are selected. This makes calibration simpler by avoiding the case that resistive losses in the supply vary depending on the calibration, and lead to changing performance as the pixel is calibrated.

Once the trim voltage has been generated, it is applied to the trim capacitor C1 using the transistors M3–M6. These are arranged so that the trim voltage can either be added to the value from the shaper (using the calib_plus path) or subtracted from it (using the calib_minus path). This has the effect of doubling the range that can be covered by the trim DAC. The advantage of this method over adding an ×16 branch to the DAC is that it requires much less space, since the M3–M6 switches are much smaller than the extra devices that would be needed for an ×16 branch. Once the trim voltage has been written onto the capacitor, the calib switches are disabled, and the operate switches are activated, placing C1 in the signal path, and adding the desired trim voltage so that it compensates the comparator offset and variation in the shaper DC level.

**Figure 8:**
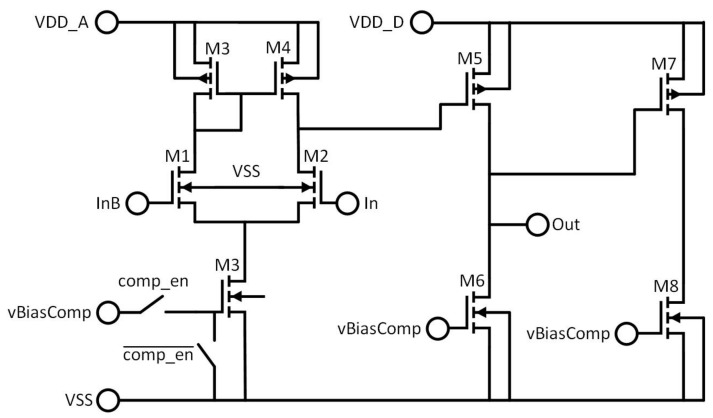
Detailed schematic of the in-pixel comparator.

The comparator itself is a simple two-stage uncompensated op-amp as shown in Figure 8. The comparator contains two complementary output branches, which help to maintain a constant current from the comparator at the cost of increased power consumption. As with the DAC, the goal of this is to ensure that resistive losses in the array power supply remain constant. Since there is still significant switching activity on these branches, they are run from a supply separate to that which supplies the more sensitive analogue circuitry. The comparator can also be powered down by disconnecting its bias voltages and pulling the gates of the bias transistors to ground. This is needed for calibration, which is further explained in Section 4.2. The same signal which disconnects the bias also disconnects the pixel from the counting bus as shown in Figure 6.

The output of the comparator clocks a D-type flip-flop whose D input is tied to the power supply. A D-type flip-flop is a memory element which copies the value at the input D to the output Q every time it detects an edge of the signal at its clock input. In the configuration described here, therefore, it acts as a memory register, copying a high logic level from D to Q when the comparator detects a hit, and holding it ready for the summation stage of the pipeline. The memory register is sampled by a global 40 MHz clock “arrayClk”. This isolates the analogue detection time of the pixel as the first step of the pipeline. Once the comparator has been sampled, the memory register is automatically reset in order to be ready for the next incoming hit.

To allow for characterisation and debugging, the chip contains a test pixel, in which the output from the shaper and comparator of that pixel can be viewed outside the chip. The test pixel is located one pixel to the left of the left hand edge, and one pixel down from the top of the chip in order to be fully surrounded by pixels and thus avoid spurious charge collection effects coming from being at the edge of the array. The shaper and comparator outputs are buffered through source followers to ensure that they can drive an oscilloscope probe or another test device correctly.

### 3.2. Column-Level Summation

Once a series of pixels has registered a hit, the number of hits in a column must be summed and provided to the periphery within the next 25 ns sampling period. Achieving this is one of the more complex parts of the design, since several competing requirements must be met:

- The sum must be completed in 25 ns;
- The silicon area required to achieve this must fit within a reasonable area of the 55 × 55 μm${}^{2}$ pixel;
- The number of vertical lines running vertically in the column that are needed to move data between pixels for summation must fit easily within the available 55 μm pitch.

Calorimetry is a niche application so there are few published attempts to meet these requirements. In [15], SRAM is used to store hits before reading them out, but this leads to an appreciable dead area in the chip. In [13], an existing chip is used. However, this make uses of a rolling shutter readout scheme (in which, once hits are recorded in the pixels, they are read out sequentially row by row). This relatively slow readout mechanism limits the event rate of the system.

A possible source of solutions from other fields are Single-Photon Avalanche Diode (SPAD)-based devices. SPADs are single-photon sensitive devices, so some form of counting solution is normally needed to count incoming photons. They are typically deployed in LiDaR, Flouresence Lifetime Imaging (FLIM) or Raman Spectrometry applications. These applications have different requirements to ours, but implement potentially useful counting solutions. In [33], for example, hits are stored in pixel, then read to the periphery, but the frame rate is limited to 24 kfps—too slow for the 25 ns target. In [34], summation for histogramming purposes is carried out in the periphery, using data from the array. However, only a single hit per column can be detected, and an anti-collision bus is used for situations with multiple hits. This would not be suitable for our requirement to count up to 15 hits per column. In [35], counting is achieved using a 14-bit counter per pixel, placed in a second layer beneath the SPAD array. This is promising, but a two-layer CMOS process was not available for our application, and in any case, the time required to read the counters leads to an overall frame rate of 30 fps, which does not meet the 25 ns requirement.

In [36], however, an interesting architecture is used. Here, SPADs are grouped, so that the total number of hits from each group can be combined. In this work, monostables are used to reduce the width of the pulse generated by each SPAD when it fires. The resulting pulses are then passed through a cascade of OR-type logic gates, leading ultimately to a single wire containing a rapid chain of pulses representing the number of SPADs that have been hit. This chain of pulses is then used like a clock to operate a counter, leading to a count of the total number of hits. The results from the various counters are then combined using a set of distributed adders. This multi-stage method of summing hits potentially offers a promising solution for the calorimetry case, but the pulse chain generation requires that the incoming signal have some distribution in time, whereas, in our case, the signals arrive in different pixels effectively simultaneously.

Therefore, a method of implementing this distributed, multi-stage summation that could handle simultaneous arrival times was sought. The approach adopted was to make use of ripple adders composed of full and half adders for all stages of the multi-stage summation. Ripple adders are a simple type of adder in which the lowest bit of of a sum is added first, then the next, and next, and so on until the sum is completed. There are essentially 3 basic ways to complete the sum using such building blocks:

- A cascade approach, in which 2 pixels are summed, then added to the next to make 4, then two 4 s combine to make 8, until 64 is reached. This uses distributed addition, but is essentially a single-level sum.
- A waterfall approach, in which pixel 63 adds to 62, to 61, to 60, and so on. As above, this is distributed addition, but on a single level.
- A mixture of the two, in which groups of pixels use the cascade approach, and the results of these sums combine in a waterfall fashion. This is a multi-stage, distributed approach, since the cascade stages proceed in parallel, and are then combined in the waterfall stage.

**Figure 9:**
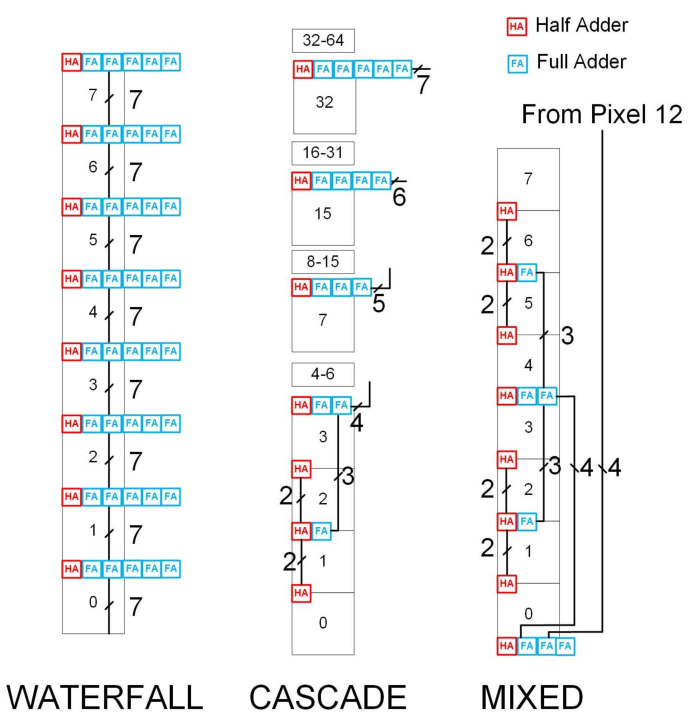
Diagram illustrating the possible options for summing a column of pixels. Numbers in boxes represent pixel numbers, other numbers show bus widths.

Figure 9 illustrates these three possible options.

It is clear that each of these options will have very different impacts on the three targets outlined previously. To determine which is the most suitable, we derive expressions for the number of gates needed, summation delay and number of parallel lines in a column required by each of these solutions. This will allow an informed choice to be made regarding the correct approach.

The basis of each method is the ripple adder, which for an output bit width *W* is composed of *W*-1 Full Adders and *W* Half Adders. A full adder contains 5 logic gates, and a half adder—2. The delay imposed by a a full adder is equivalent to 3 gate delays, and the half adder to 1. The total delay and number of gates required by a ripple adder can, therefore, be expressed as

(5) $$t_{ripple}=\left(3\left(W-2\right)+1\right)t_{gate}$$

(6) $$ngates_{ripple}=5\left(W-2\right)+2$$

where *W* is the width of the output bus and $t_{gate}$ is the propagation delay of a single gate. Looking first at the full waterfall option, the results in Equations (5) and (6) can easily be expanded to the case of a ripple adder between every pixel

(7) $$t_{waterfall}=\left(3\left(W-2\right)+1\right)\times t_{gate}\times P$$

(8) $$ngates_{waterfall}=\left(5\left(W-2\right)+2\right)\times P$$

where *P* is the number of pixels in a column. The calculation of the number of parallel lines is simple, since it is simply the width of the bus.

(9) $$nlines_{waterfall}=W$$

The calculation for the full cascade option is more complicated. First, we tackle the delay calculation. At each stage, the width of the ripple counter increases by 1 bit, and the total delay is the sum of the time required for all these ripple counters to settle. If the width of the bus is sufficient to count every pixel, the number of stages is equal to *W* − 1 and the total delay can be expressed as

(10) $$t_{cascade}=\sum_{n=0}^{W-1}\left(3n+1\right)\times t_{gate}$$

However, as we we stated earlier, for practical reasons, it may be useful to limit the size of the bus so it can handle a count less than all the pixels in a column. If we do this, then the size of the ripple counter in the cascade stops growing above this limit, and following stages remain the size of the bus. The total delay is then

(11) $$t_{cascade}=\sum_{n=0}^{W-1}\left(3n+1\right)\times t_{gate}+\left(P_{bits}-W\right)\times \left(3n+1\right)\times t_{gate}$$

where $P_{bits}$ is the number of pixels in a column expressed in bits, rounded up to the nearest bit (i.e., the smallest power of 2 that can contain the number of pixels) and we assume *W* < $P_{bits}$. Since the number of parallel lines increases in a similar way, it can be expressed similarly.

(12) $$nlines_{cascade}=2+\sum_{n=1}^{W+1}\left(n+2\right)+W$$

The formula for the number of gates appears complex, but it can be derived by inspection of Figure 9. At each stage, the number of gates can be calculated as the total number of gates in the new ripple adder added at that level, plus double the previous calculated adder (since two feed into each new level). This is represented by the first summation term in Equation (13). The final ripple adder is not doubled—it appears as the 5(W−1)+2 term. If, as before, we impose a limit on the width of the bus, the cascade stops growing above this level, but additional ripple adders with the same width as the final one must be added in order to cover the whole column. Similarly, the initial term must be multiplied by a term $\frac{P}{2^{W}}$ (the total number of pixels divided by the number covered by the small cascade) to account for the fact that the cascade did not grow to fill the whole column, so the smaller cascade must be repeated enough times to fill the column.

(13) $$ngates_{cascade}=\frac{P}{2^{W}}\left[\sum_{n=0}^{W-2}\left(2^{n}\left(5n+2\right)\right)+5\left(W-1\right)+2\right]+\sum_{n=W}^{P_{bits}}\left(5W+2\right)$$

Using these formulae, we can estimate the summation time, number of gates, and number of parallel lines needed for each option. We also illustrate the difference between a case in which we count hits coming from all 64 pixels, and one in which we only sum up to a limit of 15. Table 2 illustrates this.

The first point to note from these results is the importance of applying a counting limit. Without one, the performance of both schemes would be completely unsuitable—the waterfall scheme exceeds the 25 ns target, and the cascade scheme requires so many parallel routing lines as to be totally unworkable. Applying a counting limit eases these problems. This illustrates the importance of the background study in Figure 1.

However, even with this limit, neither scheme is ideal. The waterfall scheme is still too slow, and the 24 parallel lines of the cascade scheme still represent a considerable routing challenge. Both also have considerable degrees of freedom—we do not require a 7.2 ns summation time, or to have only 4 parallel lines. Therefore, it was decided to explore whether a mixed scheme could trade off this unnecessary performance for improvements in other areas. In a mixed scheme, the column is divided into blocks, inside each of which the cascade scheme is implemented. The separate cascade schemes are then combined using the waterfall scheme.

The key parameters of this scheme can also be expressed mathematically by combining the expressions in Equations (7)–(13). Here, we also now define a parameter *B*, which represents the size of the block into which we divide the column, and $B_{bits}$, which is this value expressed in bits and rounded up to the nearest bit as was done earlier. The expression for the number of lines required can be generated by combining the expressions for cascade and waterfall in Equations (13) and (8) and including the effect of *B*.

(14) $$nlines_{mixed}=\sum_{n=0}^{B_{bits}-2}\left(n+2\right)+W$$

Similarly, the delay time can be expressed by combining the effect of the block cascade (all blocks proceed in parallel, so only 1 must be counted) and adding it to the waterfall delay as in Equation (15). The waterfall delay is calculated as in Equation (7), but now instead of a multiplicative factor *P*, which assumed a ripple adder in every pixel, there is a factor $\frac{P}{B}$ which indicates a waterfall type ripple adder in every block.

(15) $$t_{cascade}=\sum_{n=0}^{B_{bits-1}}\left(\left(3n+1\right)\times t_{gate}\right)+\frac{P}{B}\times \left(3\left(W-2\right)+1\right)\times t_{gate}$$

Finally, the number of logic gates can again be calculated by combining those for waterfall and cascade. Once again, *P* is replaced with $\frac{P}{B}$, and the cascade expression is re-written to stop at $B_{bits}$.

(16) $$ngates_{cascade}=\frac{P}{B}\left[\sum_{n=0}^{B_{bits}-2}\left(2^{n}\left(5n+2\right)\right)+5\left(B_{bits}-1\right)+2\right]+\left(5\left(W-2\right)+2\right)\times \frac{P}{B}$$

**Figure 10:**
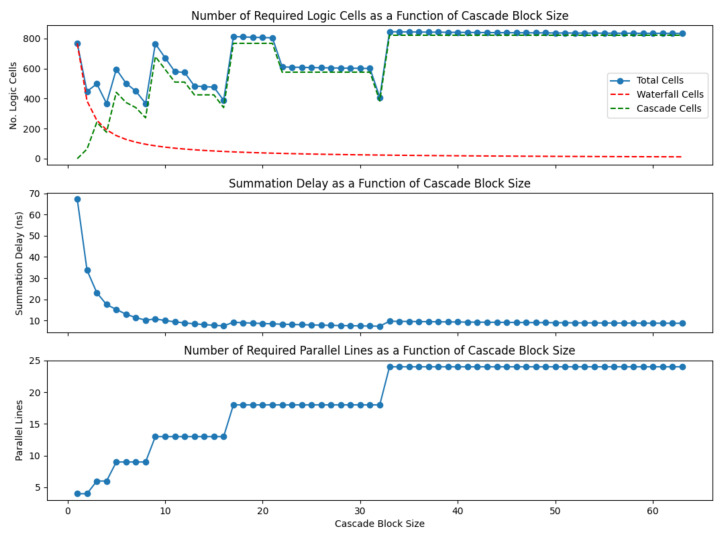
Effect on summation delay, number of parallel lines and number of logic gates of varying the size of the cascade block in mixed cascade/waterfall summation scheme.

This now leaves us with the question of what value to select for *B*, the size of the cascade block. To study this, Equations (14)–(16) were coded in Python and simulated for values of *B* between 1 and 64. The count limit of 15 was applied. Results are shown in Figure 10.

From this chart, we can see that the number of parallel lines changes from 4 to 24 as the block size changes. This is as expected, since a block size of 1 effectively corresponds to waterfall mode, and a size of 64 corresponds to cascade mode. Similarly, the summation delay has limits equivalent to the two modes. The graph illustrating the total number of logic cells also shows the expected limits at the extremes, but we can see another interesting characteristic. There are minima in the graph corresponding to block sizes equal to powers of 2. After each of these, the logic cell count jumps up, then slowly reduces again. This is because the cascade can only increase size in powers of two, and therefore, the available logic is used most efficiently when the blocks have this size. For example, for a block size of 4, the number of gates in the cascade is the minimum number which can operate a cascade of this size. If the block size increases to 5, the cascade must increase to effectively an 8 pixel cascade, meaning a large number of gates, which repeat every 5 pixels. As the block size increase to 6, 7, 8, the number of gates in the cascade does not increase, but the number of times it repeats decreases, until a local minimum at 8. This repeats for every power of 2, and the local minima have broadly similar values.

Using this information, a suitable block size was selected. It was decided to select a block size somewhere in the flat region of the Summation Delay curve, since this would avoid a situation where small variations in some parameter could lead to large changes in the summation delay—possibly leading to a value above the specification. This also helps to protect against process variation in the final chip. This being the case, it made sense to select one of the available minima in this region—either 16 or 32. Selecting 16 made most sense from the sensor layout point of view, since it requires the fewest lines—13 versus 18 for a block size of 32. Therefore, a block size of 16 was selected. Table 3 compares this option with the full waterfall and cascade options, and illustrates how the mixed option achieves a similar summation delay to the cascade, but with a significant reduction in logic and routing effort required.

**Figure 11:**
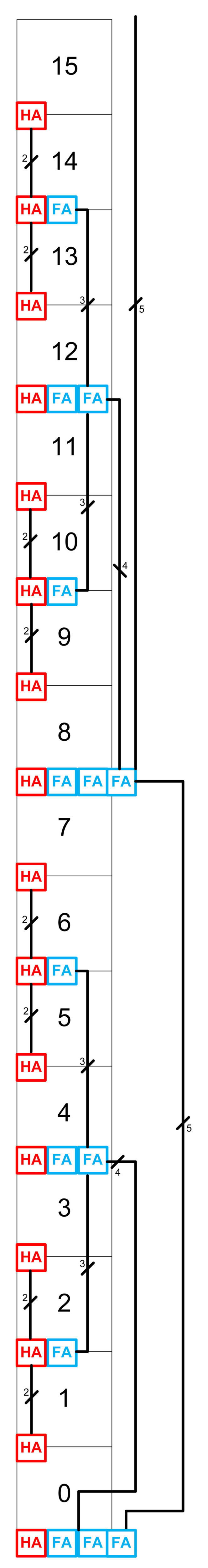
Diagram showing the 16-pixel summation scheme building block used to make a full column.

Figure 11 presenrs the final scheme for the 16-pixel block. Four of these are stacked to make a complete 64-pixel column.

Whilst this design is clearly well suited to the 64 row chip of this work, a future calorimeter would have to cover a large area. This would be achieved by tiling multiple chips, but to do this efficiently requires that individual devices be as large as possible. It would, therefore, be necessary to extend the summation scheme to its maximum feasible extent. As Table 3 shows, the current scheme is well within the 25 ns timing window for the LHC. Therefore, the design can be expanded in the first instance without any changes to the architecture simply by increasing the number of pixels up to the maximum number that can complete the sum in 25 ns. Based on the calculations in this section, the column can be expanded to a height of 320 pixels (20 × 16 pixel blocks) whilst keeping a summation time of 24.3 ns.

**Figure 12:**
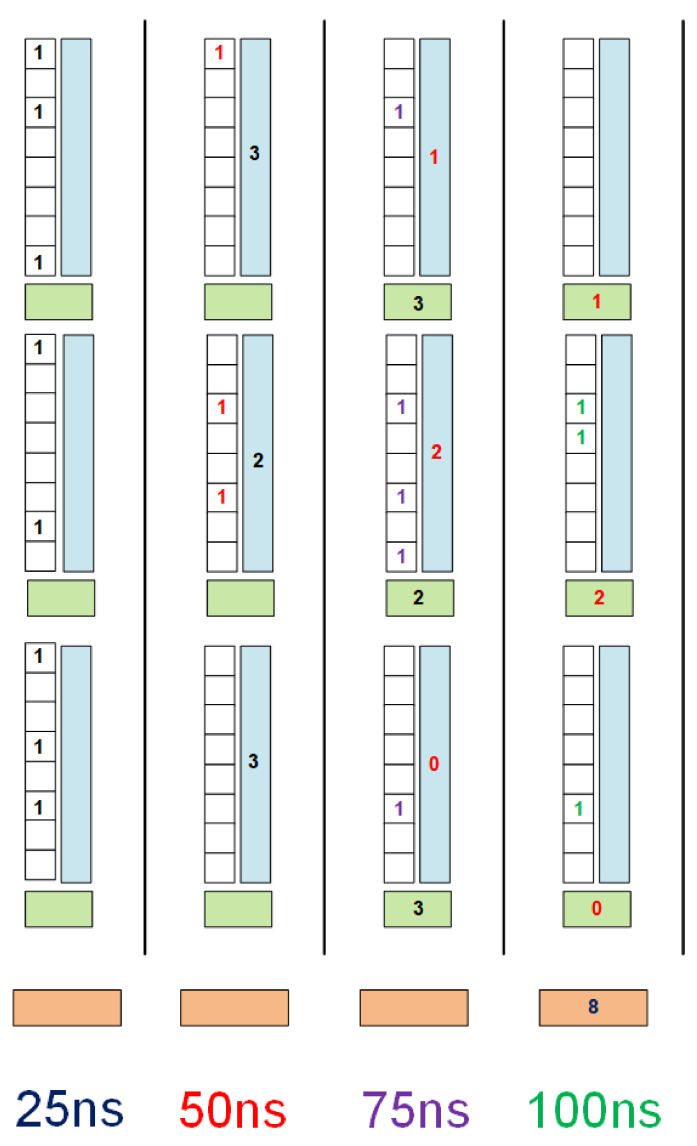
Diagram illustrating how the addition scheme could be expanded to cover a larger area. White boxes represent blocks of 320 pixels, blue boxes are the previously discussed summation scheme, and green boxes represent the additional pipeline suggested for future devices. Orange boxes indicate the peripheral logic. The different coloured numbers represent hits occurring at each of the similarly coloured time steps, and being summed as the pipeline proceeds.

However, at the existing pixel pitch of 55 μm, this still covers a height of only 17.6 mm, and a further increase in size might be desirable. This can be achieved by expanding the pipeline scheme of Figure 3 to include multiple stages in the array as illustrated in Figure 12.

Figure 12 demonstrates how an extra storage register could be added between blocks of 320 pixels to provide another 25 ns stage to the pipeline. For a sensor of 10 cm height or more, only 6 such stages would be needed, and the results stored in them could, therefore, be combined using a waterfall addition scheme. Such a scheme requires 6.3 ns for six stages, so would easily complete in this time. This comes at the cost of four additional lines, and the additional storage registers and adders needed. If these could not be fitted into the existing layout, then a row of pixels would have to be skipped every 320 in order to make space.

This describes how the sum is carried out on a per column basis, and how the scheme can be expanded to larger devices. The next section describes how all 64 columns on the chip are added together to give a pad sum, or read out individually as if they were strip sensors.

### 3.3. Periphery-Level Summation and Reconfigurability

The purpose of the peripheral circuitry is to either sum the hits and overflow bits and output two numbers (pad mode), or to provide the total number of hits for each column (strip mode). In strip mode, the total number of hits per column is limited to three to remain within the available output bandwidth.

**Figure 13:**
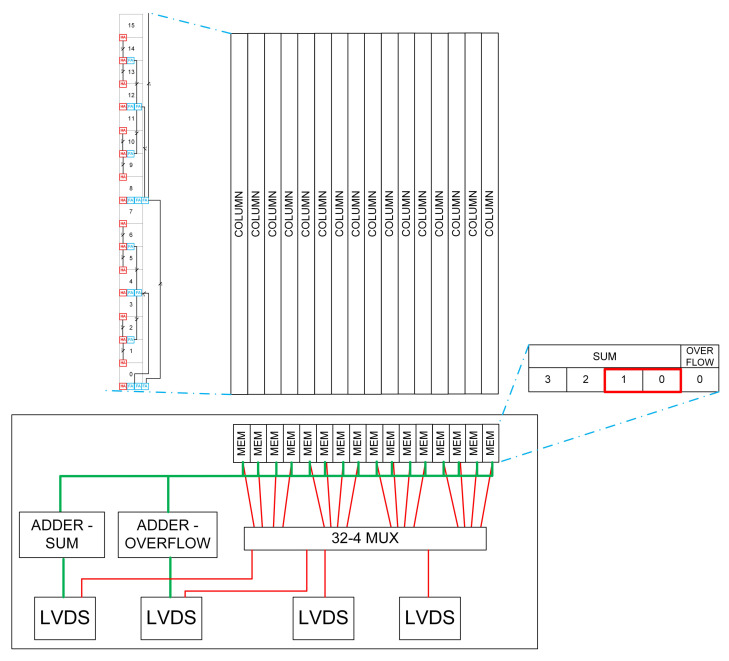
Outline of how the peripheral logic of the chip functions. The zoomed section indicates where the column from Figure 11 fits in. The green path represents “pad” mode, and the red path “column” mode.

Figure 13 shows how the peripheral logic is arranged. The chip is broken into 16 column sections, each with 4 Low-Voltage Differential Signalling (LVDS) output pads. LVDS is a standard for high-speed data transmission and LVDS output channels are selected to reduce the number of transmitters needed for the data volumes that must be transmitted. In pad mode, sums and overflows are added to give 2 total values which are transmitted as 8 bit numbers through 2 LVDS pads. In strip mode, the chip reads out each column in turn, sending out 2 bits for each, representing 0, 1, 2, or many hits. This mode requires all four LVDS pads for data transmission.

**Figure 14:**
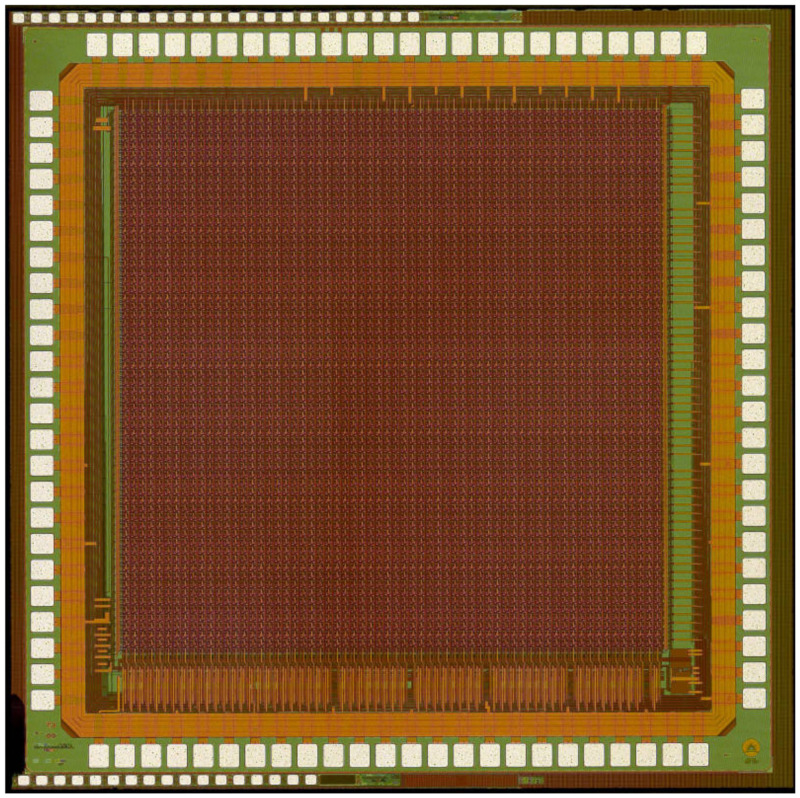
Photograph of the completed chip.

The chip was fabricated in a 180 nm CMOS Image Sensor process. A photograph of the completed chip is shown in Figure 14.

## 4. Testing and Performance

**Figure 15:**
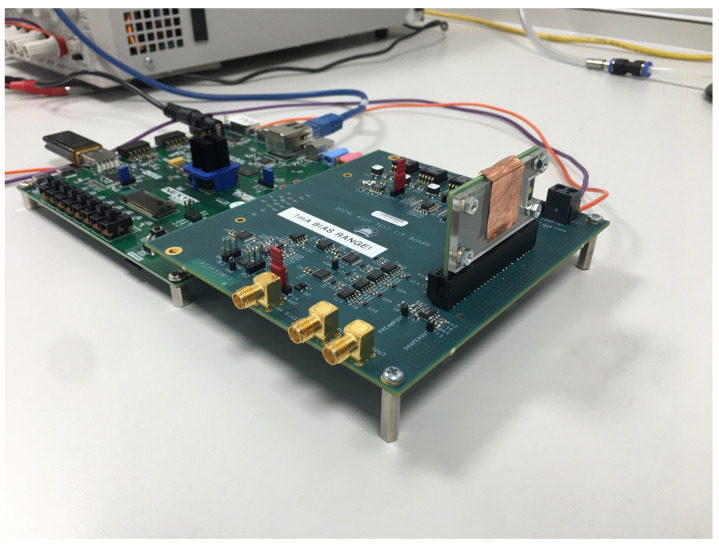
DECAL mounted on its daughterboard and installed in the test system.

A supporting test system for the sensor has been designed, and testing is underway. The system consists of a daughterboard to which the chip is bonded, supported by a motherboard providing power and bias resources. The assembly is operated by a commercially available FPGA board. The system is more fully described in [37], and is pictured in Figure 15.

### 4.1. Previous Results

Some considerable testing of the chip has already been performed. In [38], full functionality of the summation circuitry at the design speed was demonstrated by injecting data from the test register. This showed that both pad and strip mode functioned as expected, and the overflow bit was triggered at the expected point. In [37], first, the analogue output of the test pixel was examined. This demonstrated that the response of the shaper and comparator to laser stimuli was a good match to the expected performance from simulations. The authors of [37] also extended the testing of the summation circuitry performed in [38] by sweeping the pixel threshold, and observing that the count of the number hits in pad (pad mode) or strip (strip mode) rose in the expected manner. This demonstrates that the pixel and summation scheme are operating correctly together, and the chip is capable of counting pixel hits in the required manner.

The work in [39] expanded this testing of the chip’s ability to count hits further by using a laser and a grid of differently shaped holes to illuminate different sized areas of the sensor while keeping the threshold level constant. This demonstrated that the ability of the chip to count varying numbers of hits functioned correctly. The authors of [39] also conducted more detailed testing of the analogue performance of the pixel, this time using the full matrix, and a copper $K_{\alpha }$ X-ray source which generated a signal of approximately 2440 $e^{-}$- roughly equivalent to a Minimum Ionising Particle (MIP). The threshold was scanned to evaluate the noise and the measured signal size. These tests demonstrated an average sensor noise of 2.5 mV and signal of 68 mV. Taken together with the size of the X-ray signal, this implies a conversion gain of 30 μV/$e^{-}$ and a noise of 82 $e^{-}$. Whilst this is the average, this testing also demonstrated considerable variation in both values across strips. This is likely due to variation in the conversion gain coming from the very small input capacitance. This is supported by the fact that signal and noise tend to move in the same direction from pixel to pixel. The end result of this is that in spite of the variation, the Signal-to-Noise (SNR) ratio for each strip is never less than 10, making the chip suitable for MIP detection.

The previously cited papers were working with the first version of the chip in which a design error in the clock tree made it possible to program the calibration DAC correctly only in every other pixel. To correct this, a new version of the chip was produced and testing of this is described in [40]. This chip was also fabricated on a new process which aims at full depletion of the epitaxial layer for improved radiation hardness [41]. These modifications permitted full use of the bias DAC, and threshold uniformity of <1 mV over 85% of the array was demonstrated. X-ray calibration was also repeated on the new device, and a ${}^{90}$Sr source was used to demonstrate MIP sensitivity, although it was observed that further improvements to the triggering of the DAQ system would be needed to fully characterise the chip using this isotope. A conversion gain of 18 μV/$e^{-}$ was reported. This is lower than previous results, and could be a result of the different silicon processing used.

### 4.2. Mask Register Testing

As the calibration register of the chip is now fully functional, it is possible to obtain full access to the mask bit in every pixel. The purpose of this masking is to allow certain pixels to be turned off for calibration reasons. This is needed for two reasons. Firstly, since the readout of the chip is columnar in nature, it is necessary to disable all but one pixel in a column when calibrating, in order to have access to the data from only the pixel being calibrated. Secondly, the chip is designed for operation in a low occupancy environment, i.e., one in which few hits occur over the area of the detector. However, calibration is performed by placing the threshold close to the noise floor of the chip—this leads to a huge number of hits, which causes a high current draw, a drop in the power supply due to resistive losses, and a resultant drop in performance of the chip. Masking is, therefore, used to prevent the comparator firing, and the resulting reduction in the supply level affecting the calibration. Rotating the mask allows all pixels to be characterised in turn.

**Figure 16:**
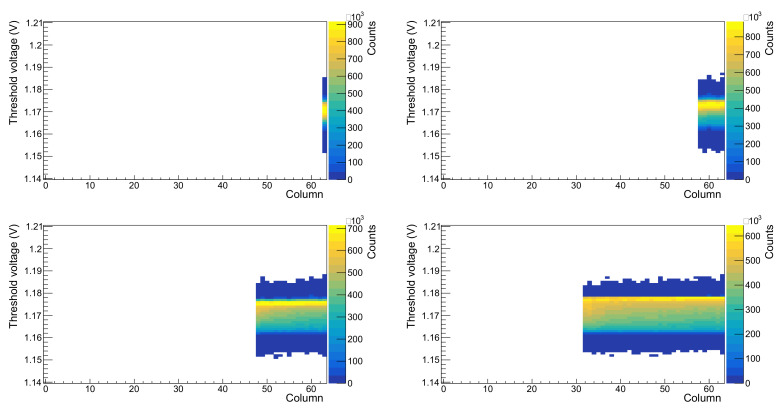

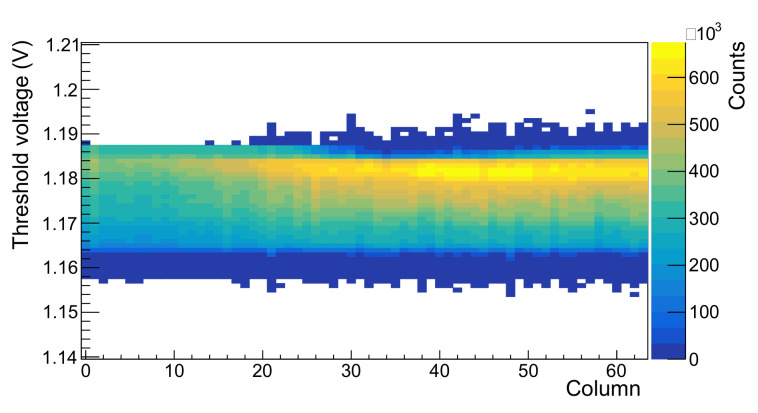
Test of the DECAL mask register. Test is a threshold scan with variable numbers of strips of pixels masked. Reading top to bottom, left to right, the number of unmasked strips is 1, 6, 16, 32, 64.

The mask register allows any combination of pixels to be disabled. This was tested in various combinations, and Figure 16 shows one example.

As can be seen from these results, the masking is also working effectively, and strips can be excluded from contributing to the overall count in the expected manner.

### 4.3. Testing with Americium-241

**Figure 17:**
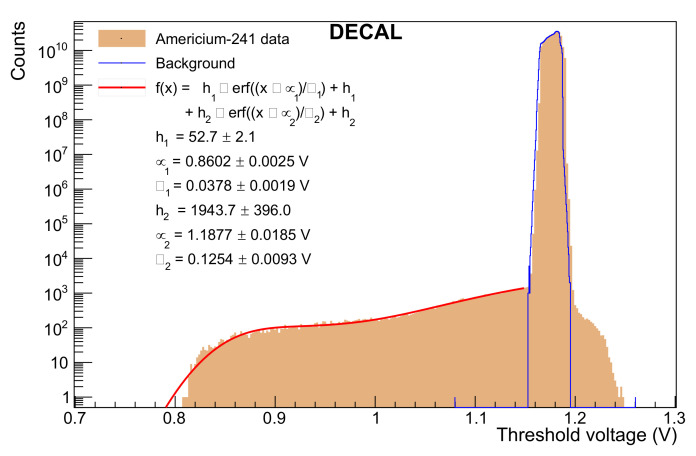

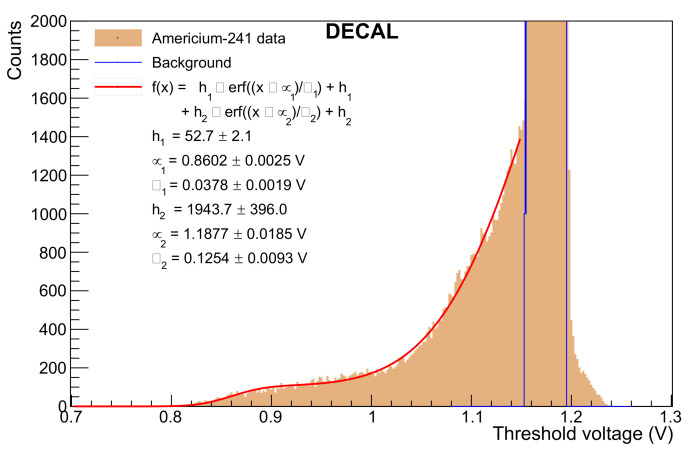
Full data from ${}^{241}\mathrm{Am}$ source test. The top plot shows the full histogram from the source test. The lower plot is a zoomed-in view of the signal shoulder on linear scale. The height $h_{i}$ is the multiplicative constant of the corresponding error function and the mean $\mu_{i}$ and width $\sigma_{i}$ its parameters, obtained by the red fit function to the signal shoulder. Background data measured without the Americium source incident on the sensor are indicated by the blue line.

The next stage of testing used ${}^{241}\mathrm{Am}$ to characterise the chip. ${}^{241}\mathrm{Am}$ decays into ${}^{237}\mathrm{Np}$ via alpha decay and populates an excited state of 59.54 keV in 84.5 % of the decays. Subsequent de-excitation happens via a photon emission of the same energy. Figure 17 shows a histogram resulting from scanning the threshold voltage whilst an ${}^{241}\mathrm{Am}$ source is placed in front of the DECAL sensor. In this case, all pixels are unmasked to generate the maximum possible number of counts, leading to a higher background noise than previously discussed. This comes from the power supply effects mentioned in Section 4.2, and would not be present for a normal operational threshold, or were masking employed. A signal shoulder stands out from the background measurement and can be described by the sum of two error functions. This fit function is motivated by the threshold counting behaviour of the sensor. A threshold scan measures the integrated energy spectrum, meaning that a Gaussian energy profile leads to an error function in a threshold scan. The error function centered at $\mu_{1}=0.860$ V describes the 59.54 keV photon emission and lies 320 mV below the baseline of 1.180 V. The baseline is determined by the threshold voltage where we find most hits due to random noise voltage fluctuations taken from the background data. It is noted that in 25 μm silicon the photon absorption probability is only 0.19% at 59.5 keV while at 10 keV it is two orders of magnitude higher as it evaluates to 17.90% [42]. A second error function was chosen to enfold the low-energy photon emissions that happen with lower probability but preferred absorption probability.

### 4.4. Testing with Cu and Mo X-ray Targets

**Figure 18:**
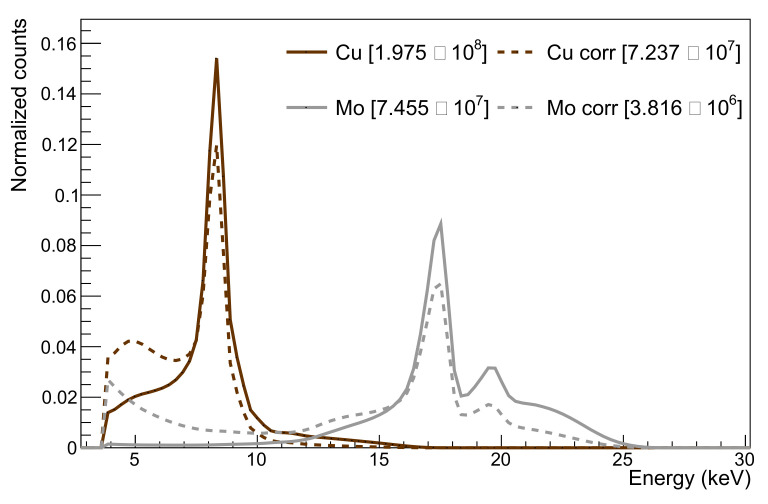
Copper and molybdenum X-ray fluorescence spectra measured with the HEXITEC detector (solid) and corrected for the energy dependent photon absorption probabilities in 25 μm silicon (dashed). All spectra are normalized by the corresponding constants stated in the legend.

Given the low interaction probability in the previous tests, further testing was undertaken at lower photon energies using the X-ray fluorescence of copper and molybdenum targets. The $K_{\alpha }$ excitations of these targets lie at 8.05 and 17.48 keV, respectively. Their energy spectra are measured with the HEXITEC detector manufactured at RAL [43] and then corrected for the energy-dependent photon absorption probabilities in 25 μm silicon. The nominal spectra as well as the spectra after folding in the absorption probability are shown in Figure 18.

**Figure 19:**
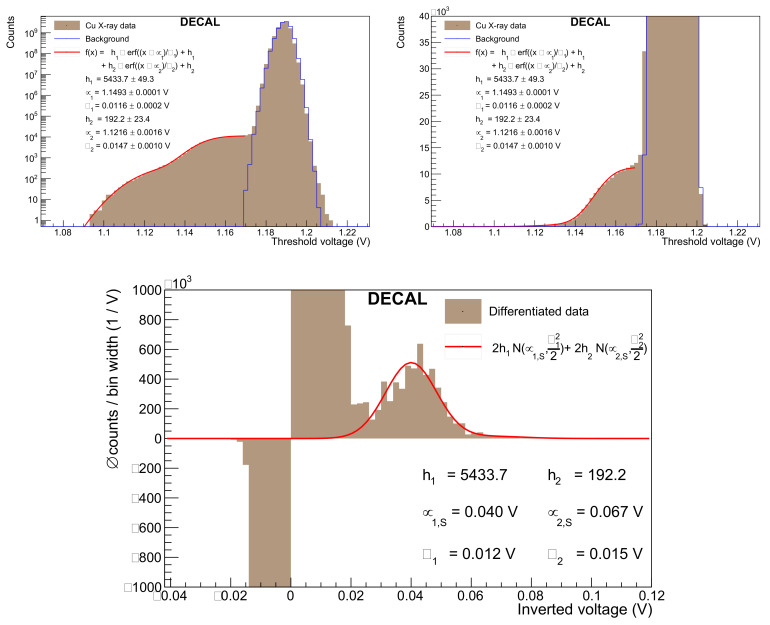
Threshold scan with the DECAL chip under Cu X-ray fluorescence on logarithmic (**top left**) and linear scale (**top right).** The **bottom** plot shows the binwise differentiated data on an inverted x-axis whose origin is defined to be the baseline voltage. The numerical differentiation is the difference in counts between neighbouring threshold scan bins divided by their width of 2 mV. The fit function is analytically differentiated and two Gaussians are obtained. Background data measured when the X-ray source is turned off are indicated by the blue line.

**Figure 20:**
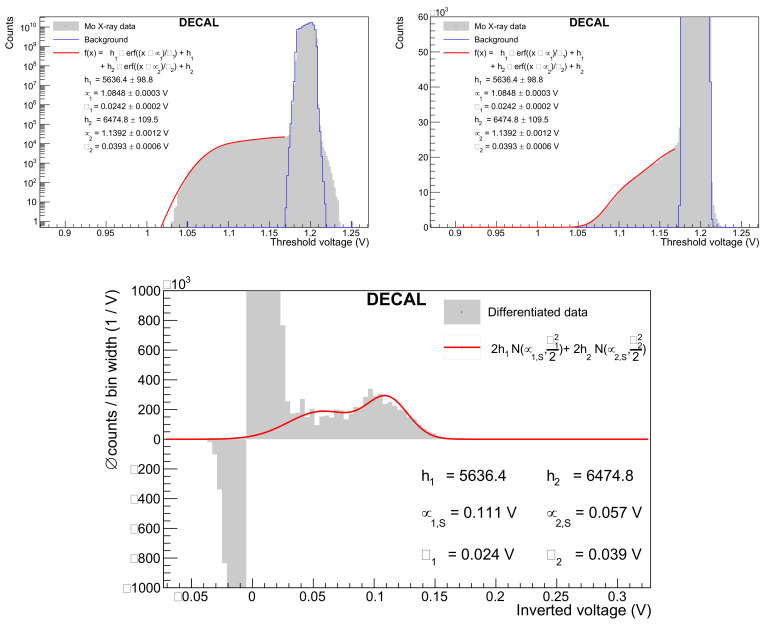
Threshold scan with the DECAL chip under Mo X-ray fluorescence on logarithmic (**top left**) and linear scale (**top right**) and their differentiation (**bottom**). The differentiation of the threshold scan data is here performed with a bin width of 4 mV due to the larger threshold voltage range. Background data measured when the X-ray source is turned off are indicated by the blue line.

A DECAL threshold scan for copper X-ray luminescence is performed with all but one row masked, and it is shown in Figure 19 on logarithmic and zoomed-in linear scale together with a fitted double error function. The differentiated data and fit function are additionally plotted on an inverted x-axis by plotting the difference between the baseline voltage and the tested threshold voltage. Such a spectrum can be understood as an energy spectrum. The peak in that spectrum corresponds to the Cu $K_{\alpha }$ excitation and is centred at a signal height $\mu_{1,S}$ of 40 mV. For molybdenum X-ray fluorescence, the DECAL chip was measured with no masked rows. Following the same analysis steps as for the copper data results in a separation of 111 mV between the baseline voltage and the mean of the error function. This signal height is extracted from the differentiated spectrum in Figure 20.

The energy for an electron–hole pair creation of ϵ=3.6 eV enters the conversion gain calculation. In the case of Mo $K_{\alpha }$ fluorescence, the number of produced electron–hole pairs is assumed to be 17.48keV/ϵ=4856. Dividing the signal height by the number of electrons leads to a conversion gain of 22.9 μV/e^−^. As an alternative signal height estimator, the position of the peak maximum in the differentiated spectrum is considered. For molybdenum, the signal height for the maximum value reduces to 97 mV and the conversion gain accordingly to 20.0 μV/e^−^. Table 4 further states the gain obtained through the americium and copper measurements. The standard deviation of the background data is lower for copper because all but one row was masked.

## 5. Discussion

In this paper, we have described the DECAL sensor, a MAPS device intended to demonstrate new ways of performing digital calorimetry in future colliders. We have outlined how the device operates in pad mode for calorimetry, and in strip mode, so that the same device can be re-configured to operate as a strip tracking chip in outer tracking and pre-shower layers. The theoretical basis for the design has been outlined, and the operation explained.

Previous test results taken using this sensor have been summarised, and the results of recent tests have been reported. These results demonstrate that, on top of the previously demonstrated performance, the masking function is fully operational. Furthermore, additional tests using ${}^{241}$Am, and X-ray fluorescence from copper and molybdenum targets establishes the conversion gain as approximately 20 μV/e^−^ (taken as the average of all measured results), in line with previous results and expected performance.

Future work is likely to focus on establishing the effect of the new processing on the device’s performance, and further detailed characterisation. In terms of chip development, a next generation of the device could include several improvements, such as a low power front-end, on-chip data compression or the addition of a “pixel” readout mode to complement the existing “strip” and “pad” modes. This would allow re-use of the device in almost all areas of a future collider. These improvements would be aimed at making the integration of the device in future experiments simpler.

## Figures and Tables

**Table 1:** DECAL Specification.

Specification	Unit	Value
Pixel Pitch	μm	55
Resolution	pix	64 × 64
Frame Rate	MHz	40
Input Referred Noise	$e^{-}$	80
Max hits/col (pad mode)	hits	15
Max hits/col (column mode)	hits	3
Other features	N/A	Overflow indication, masking, test register

**Table 2:** Resources required for cascade and waterfall summation schemes.

Scheme Counting Limit	Waterfall		Cascade	
	64	15	64	15
Summation Time (ns)	124.8	67.2	7.7	7.2
Number of Gates	1408	768	854	839
Number of Parallel Lines	6	4	41	24

**Table 3:** Resources required for cascade, waterfall and mixed summation schemes.

Scheme Counting Limit	Waterfall		Cascade		Mixed—Block Size 16
	64	15	64	15	15
Summation Time (ns)	124.8	67.2	7.7	7.2	7.5
Number of Gates	1408	768	854	839	388
Number of Parallel Lines	6	4	41	24	13

**Table 4:** Signal height and conversion gain for different X-ray energies and photons from Am decay. The signal height is either extracted as the mean of an error function fit or the position of the maximum differentiation value. The method is marked accordingly. σ is the standard deviation of the background noise peak.

Source	Photon Energy (keV)	Signal Height (mV)	Determination Method	σ (mV)	Conversion Gain (μV/e${}^{-}$)
${}^{241}\mathrm{Am}$ decay	59.54	320	$\mu_{1,S}$ from fit	5.9	19.3
Mo $K_{\alpha }$	17.48	111	$\mu_{1,S}$ from fit	6.3	22.9
Mo $K_{\alpha }$	17.48	97	max	6.3	20.0
Cu $K_{\alpha }$	8.05	40	$\mu_{1,S}$ from fit	2.6	17.9
Cu $K_{\alpha }$	8.05	43	max	2.6	19.2

## Data Availability

Not applicable.

## Abbreviations

The following abbreviations are used in this manuscript:

LHC	Large Hadron Collider
ASIC	Application Specific Integrated Circuit
MIP	Minimum Ionising Particle
DAC	Digital-to-Analogue Converter
LVDS	Low-Voltage Differential Signalling
CMOS	Complementary Metal Oxide Semiconductor
FPGA	Field-Programmable Gate Array
CMOS	Complementary Metal Oxide Semiconductor
SNR	Signal-to-Noise Ratio
DAQ	Data Acquisition
SPAD	Single-Photon Avalanche Diode
HEXITEC	High-energy X-ray imaging technology
SRAM	Static Random Access Memory
LVDS	Low-Voltage Differential Signalling
